# Design of Assessment Judging Model for Physical Education Professional Skills Course Based on Convolutional Neural Network and Few-Shot Learning

**DOI:** 10.1155/2022/7548256

**Published:** 2022-05-28

**Authors:** Qingjie Chen, Minkai Dong

**Affiliations:** ^1^School of Sports and Health, Linyi University, Linyi 276000, Shandong, China; ^2^Postdoctoral Mobile Station of Shandong University, Jinan 250100, Shandong, China; ^3^Physical Education Department, Shanghai University of Finance and Economics, Yangpu 200433, Shanghai, China

## Abstract

In recent years, the promotion of quality education and the development of curriculum and teaching materials reform have put forward higher goals and requirements for professional skills in physical education. However, there are still many shortcomings in the nuclear assessment of physical education professional skills, such as lack of clarity in evaluation objectives, lack of scientific in evaluation indexes, lack of systematization in evaluation contents, lack of diversity in evaluation methods, lack of authority in evaluation results, and lack of timely prediction and analysis of students' mastery of classroom teaching skills, thus not giving good play to all the functions that the nuclear assessment should have, thus to a certain extent fettering the further enhancement of physical education. With the development of information technology, artificial intelligence, as a new technology, can guide the improvement of the assessment and judging mode of physical education professional skills courses, and is also an important guiding idea for physical education majors to meet the development demands of information-based society. Based on the analysis of the connotation and characteristics of deep learning, this paper points out the insufficiency of the assessment and evaluation of traditional physical education professional skills courses and proposes a method of assessment and evaluation of physical education professional skills courses based on convolutional neural networks and small sample learning. In the case of a small amount of data in the course assessment, we use a small number of samples to learn, and only need a small number of samples to learn quickly. Using the improvement measures under the teaching concept of deep learning, physical education personnel are required to truly change in terms of professional skills mastery and evaluation. We effectively implement improvement measures, promote the improvement of physical education professional skills, and realize the migration and innovation of sports knowledge and skills.

## 1. Introduction

With China's increased support for the construction and development of applied higher education, the goal of higher education personnel cultivation has been rapidly changing from professional academic and theoretical research-oriented to knowledge, skill, and comprehensive ability quality personnel cultivation [1–3]. As a very important part of this professional skills education, the skills courses of physical education majors in colleges and universities assume the important task of cultivating comprehensive ability-oriented technical talents who meet the essential attributes of modern higher vocational education and meet the needs of modern society. How to accurately assess and judge the skills courses of physical education majors is an urgent problem to be solved.

**Figure 1:**
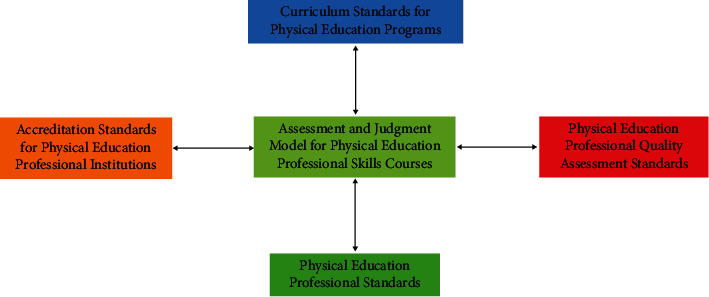
Physical education professional skills course assessment standards.

The concept of competency-based education advocates the existence of an integrated curriculum combination with competency as the basis of vocational education system and the knowledge, skills, and attitude needs of the chosen occupation as the target, formulating quantitative standards of competency for each socio-professional position, then dividing it into several small modules of levels, and then developing and teaching the competency development and implementation of skills courses as well as comprehensive evaluation. This will ultimately improve the students' comprehensive ability to engage in the profession, increase the employment rate, better meet the society's demand for talents of the profession, and better help realize the essential attributes of higher education. The principles of the construction of the evaluation system of skills courses under the competency-based approach include as follows: according to the milestones and tasks of human social competence development, the specific practical experience and evaluation of the taught group, and the professional theoretical basis of physical education teachers [4]. A comprehensive evaluation system is designed with each ability module as a unit evaluation standard. The taught group can selectively carry out a variety of independent, inquiry, interchangeable, and situational learning methods around the competence standards of their chosen technical items [5–8]. He has the advantage of being familiar with the chosen professional technical movements and completing the first step of social practice verification. The following principles have always been adhered to in the competency-based evaluation system of modern implementation. First, in order to ensure the validity and fairness and rationality of each skill evaluation index in the physical education professional skills course assessment, more forms of evaluation means will be used to the maximum extent (such as professional skills multipoint test, teaching project simulation exercises, interview impromptu questions and answers, open-book comprehensive ability theory assessment and direct observation of the taught object, and reference to its original learning evidence and other forms); second, for the current evaluation units common to the current physical skills category, the most suitable, closest, and relevant evaluation pathway is selected; finally, each time in the technical evaluation or testing, more related multiple ability units are involved, rather than for individual one or two ability units, while simultaneous evaluation and testing are encouraged. Therefore, the diversified and comprehensive evaluation model has the advantages of being holistic, flexible, scientific, and standardized compared to the evaluation methods that only focus on technical teaching tasks. The assessment criteria for physical education professional skills courses are shown in Figure 1.

Physical education is the basis of the whole education and is a complex system of functions, which gradually becomes a hot topic of public concern [9]. From the school's point of view, physical education in school is the basic education for people to understand the simple knowledge of physical health, to grasp the skills of sports, and to realize the healthy growth of students' bodies. In the context of the new educational reform, physical education teachers are finding it more and more difficult to teach physical education, revealing many shortcomings of the physical education teacher training system. The demand for professionalism of physical education teachers is getting higher and higher, and schools are putting forward higher requirements for the overall quality of physical education teachers. In order to achieve the professionalization of physical education teachers, “what to teach” and “how to teach” have become the two main issues today, and the training of physical education teachers' teaching skills is now the top priority in the training of physical education teachers and the promotion of physical education teachers' professionalization. Scholars and experts have begun to pay attention to the most basic teaching skills that physical education teachers should have [10, 11]. In recent years, the discussion on physical education teaching skills has become more and more intense, but ultimately what is the professional teaching skills of physical education and what kind of physical education skills are needed to meet the needs of physical education in schools has become an ambiguous issue, the quality of teaching has physical education teaching skills to influence, and physical education is also an important means to promote the overall quality and healthy growth of students. A qualified physical education teacher must be proficient in basic professional skills. Therefore, it is necessary to update physical education teaching skills and improve teaching skills.

Artificial intelligence technology is an information technology established on the basis of computer technology, which has strong characteristics of openness, flexibility, interactivity, and collaboration, which is conducive to enriching the content of physical education and achieving diversified development of physical education forms. The auxiliary teaching of artificial intelligence in physical education needs to carry out various teaching activities with the support of computers, and teachers can reasonably arrange the teaching process as well as the forms and techniques of sports by discussing the teaching projects in the classroom with students. Deep learning in artificial intelligence is mainly used for intelligent classification and prediction. At present, deep learning is mostly applied in the field of competitive sports, and its impact on physical education is mainly reflected in two aspects. First, deep learning algorithms can be used for sports activity type identification. For example, artificial neural networks can assess individual exercise metabolic equivalents and identify individual activity types (low-intensity activity, sports, vigorous exercise, and domestic/other activities) [12–14]; computational modeling can monitor and provide feedback on muscle status in real time, predict fatigue, and avoid sports injuries. Secondly, deep learning algorithms can be used for physical education learning diagnosis and performance prediction, by mining historical data of training and competition to predict competition results and provide data basis for tiered physical education teaching. Therefore, in order to make up for the above-mentioned deficiencies in the process of detecting wrong actions in physical education professional skills course assessment, and to address the existing physical education professional skills course assessment judging model is not enough to reflect the latest physical education needs and development, and at the same time the problem of few-shots in physical education professional skills [15] course assessment, this paper proposes a physical education professional skills course assessment judging based on convolutional neural network and few-shot learning. The method uses deep learning to improve the accuracy of the course assessment judgment, and the effectiveness of the method is demonstrated in the relevant dataset. The contributions of this paper are summarized as follows: 1. sports learning diagnosis requires high accuracy and accuracy of deep learning algorithm, and its accuracy needs to be optimized. In order to achieve the goal of error action detection in physical education professional skills course assessment, two-dimensional wavelet packet [16] technology and spatial clustering [17] technology are used to analyze human behavioral actions by parsing images in physical education professional skills course assessment to improve the accuracy of error action detection, but the features extracted by the two-dimensional wavelet packet [16] technology are not comprehensive enough, so that the subsequent detection results have a large deviation from the actual, and with the further development of related fields, deep convolutional neural networks have been widely used in the process of human action recognition and target detection, but due to the poor hierarchical nature of the constructed deep convolutional neural networks and the failure to achieve further processing of action samples. However, the applicability of this method to the detection of wrong actions in the assessment of physical education professional skills courses is poor due to the poor hierarchical nature of the deep convolutional neural network and the failure to further process the action samples. 3. A method based on convolutional neural network and few-shot learning is proposed to judge the assessment of physical education professional skills courses, and the effectiveness of the method is demonstrated in the relevant database.

## 2. Related Work

### 2.1. Physical Education Professional Skills Assessment Content

**Figure 2:**
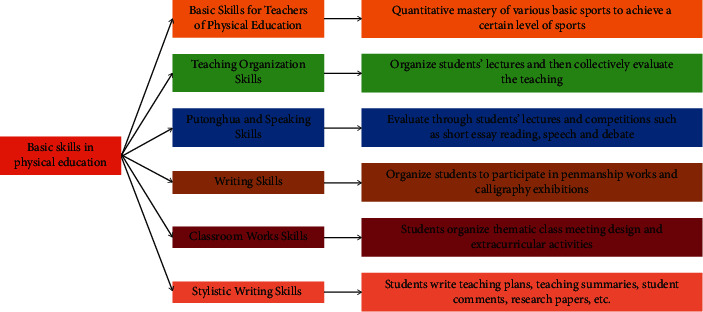
Physical education professional skills course assessment content.

The assessment content of physical education professional skills courses is shown in Figure 2. Professional basic theoretical knowledge is an important part of assessing students' foundation, which includes two parts: basic theory and professional theory, accounting for 20% of the total grade, of which basic theory accounts for 10% and professional theory accounts for 10%. The assessment process in the basic theory can be carried out by combining 3 elements of paper-based written test and interview with sample questions and application exercises. Each accounts for a different percentage [18–20]. Among them, in the written part of the paper can be used open paper (relatively broad knowledge, focusing on the practical application of its technology assessment) or closed-book (test questions are simpler, focusing on the basics) form, this item is now used more in the teaching process, but with the guidance of the competence-based, selected question interview is also increasingly used at the same time, the assessment of the staff taught on the spot to draw the answer card, requiring a short time to prepare, 10 min to carry out. The test is prepared in a short period of time, and an oral test is conducted in 10 minutes. In addition, it can also be assigned according to the accompanying exercises arranged after each class, or a large assignment of propositional argumentation [16]. Various forms of theoretical assessment have become another development direction of education nowadays. Physical education professional skills assessment content and evaluation methods are another important part of the teaching of artistic and technical skills courses in physical education. Gymnastics, sparring, artistic gymnastics, and other skill-driven difficult beauty class projects mostly use two forms of assessment: they are 1-min short time independent creation of the routine (accounting for 20%), the prescribed routine (accounting for 20%); and fencing, water polo, goalball, and other technical combat energy-driven class projects use three forms of assessment: they are the prescribed technical action assessment (15%), self-selected action (accounting for 15%), and practice (10), as such, weightlifting, marathon, and other physical dominated projects also use two forms of assessment, and they are self-selected items (accounting for 20%) and achievement (accounting for 20%). So, the taught population will be able to make a reasonable choice according to different evaluation scores and their own special ability specialties, thus promoting the improvement of performance, and also greatly increasing the personalized learning atmosphere and interest of students. In the professional sports technology assessment and evaluation, generally we will use the teaching and examination separated from each other form; that is, the teacher of the class is not responsible for the class assessment teacher and can form a scoring team for the assessment at the time of the competition and take the comprehensive results of more than 5 teachers as the final score of their assessment, so that students will be on the same starting line with the same scale, and the results will be more objective, for the taught people. It is also another aspect of stimulation for the taught, increasing the pressure of training, while turning pressure into motivation, prompting them to practice harder to achieve better teaching results. However, in some items, we can give them some opportunities for technical evaluation, for example, in the single item of athletics, and we can take the best result of their three tests as the final evaluation result. In terms of assessment time, parallel classes should arrange a unified time, place, judge, and standard for assessment. Practical ability assessment content and evaluation method, the professional skills class (art) course's practical ability assessment, and evaluation in the total grade accounted for 20%, because he must be under the premise of practical conditions to assess and evaluate, so compared to other parts of the assessment will be slightly more complex. It is better to evaluate the practical participation of the participants in the different levels of the event, mainly by observing their performance in the practical process or by quizzes or setting some small tests. This can be arranged according to the characteristics of each project. But the general principle is that the evaluation should be presented as a whole process. The methods and approaches should be flexible, and the evaluation accounts for a total of 20% of the total grade of which 10% is for in-class practice and 10% is for out-of-class practice, and the process evaluation and quantitative evaluation of the practice are also added. Ultimately, it achieves a fair and reasonable response to the level of the real practice proctoring of the taught population. The content and evaluation method of the usual performance assessment and the usual assessment of the professional skills (art) course is a comprehensive reflection of their state in the whole learning process of the course, accounting for 10% of the total grade, which is a specific evaluation of the seriousness, team consciousness, and performance of the taught group in the learning process. Among them, the degree of conscientiousness accounts for 2%, team consciousness accounts for 3%, performance accounts for 2%, and attendance accounts for 3%. Physical education quality assessment is divided into two parts: general physical education quality assessment and specific physical education quality assessment. A quality assessment of physical education accounts for 50% of the total score. And they are all achieved by using quantitative testing methods, which better reflects the fairness to the taught people.

### 2.2. Image Processing and Recognition Algorithms

Artificial intelligence is a product of the in-depth development of computer science, which has revolutionized the manufacturing of traditional electrified machinery by combining intelligent programs and high-tech chip manufacturing with intelligent robot manufacturing. Of course, artificial intelligence is not only as simple as the intelligence of traditional equipment, this is only a shallow application of artificial intelligence, and in the context of the continuous development of computer science, artificial intelligence will undoubtedly further develop to the depths of intelligence [17]. As a branch of computer science, artificial intelligence to research and development of humanized mechanical intelligence (including human action ability, cognitive ability, and thinking ability) as the core, the first through the imitation of humanized intelligence and expansion, lay the basic direction of the development of mechanical intelligence, based on this in-depth research and development, and ultimately achieve the goal of making the degree of mechanical intelligence beyond the human [16, 17, 21]. At present, artificial intelligence technology has been applied to many fields of social production and life, including intelligent robot manufacturing, the development and application of intelligent sensor identification system, digital intelligent payment technology, the development and application of intelligent database, and the development and application of intelligent network security defense system. However, in the field of culture and education, although schools at all levels have generally introduced many multimedia devices for networked teaching, the application of artificial intelligence technology is still in the shade. Whether it is mature artificial intelligence devices or digital forms of artificial intelligence technology have not yet entered the field of school education and teaching. In fact, with the mature development of AI technology, it has great potential for application in the field of culture and education, not only in replacing the role of “teachers” with intelligent robots to impart knowledge, but also in the design and application of teaching systems in schools, so that the teaching system of schools can evolve from the traditional. This is of positive significance for improving the management level of school teaching services and thus the efficiency of teaching. In this paper, we intend to take the teaching of physical education as an example and briefly discuss the application of artificial intelligence in the design of physical education teaching system, in order to provide some reference experience for improving the scientific and systematic nature of physical education in schools and improving the effectiveness of physical education in schools.

As a relatively special subject in the teaching system of university, physical education is different from the teaching of general professional theory courses. Modern physical education focuses on the appropriate and efficient coordination of theoretical learning and sports training, without overly emphasizing one or the other, and requires teachers to be able to flexibly arrange course contents and adopt effective physical education teaching methods according to the general requirements and implementation principles of modern school physical education. At present, universities or primary and secondary schools in the compulsory education stage pay relatively little attention to physical education, and physical education is often affected by other “proper subjects” and “professional courses,” and the originally small physical education The few physical education courses are dominated by other courses, resulting in a situation where students have low physical education ability and health quality. Under such circumstances, students' basic physical education needs are not met and most of the physical education policies are not implemented, not to mention the scientific and efficient physical education system to support physical education practice.

### 2.3. Few-Shot Learning Related Studies

In recent years, artificial intelligence technology has witnessed rapid development in the era of big data, rapidly transforming from early academic exploration to practical application [22]. At present, AI algorithms represented by deep learning have achieved advanced results and successfully landed in fields such as image classification, biometric recognition, and medical-aided diagnosis by virtue of ultra-large-scale datasets and powerful computing resources. However, today's real-world scenarios usually do not have access to large-scale trainable data, which are detrimental to the intelligent transformation of many traditional industries. On the other hand, since image classification algorithms play a key role in practical applications, the research of key algorithms for small-sample learning of image classification has become one of the driving engines for the intelligent transformation of industries. Deep learning is predicated on large-scale datasets and has achieved remarkable success in areas such as image classification, target detection, and text analysis. However, in practical scenarios, firstly, it is difficult or impossible for relevant researchers to obtain large-scale high-quality data and annotation due to cost, privacy, security, or ethical issues [23]. For example, in the medical field, medical images are generated from cases, but a small number of cases are not able to assist the machine to analyze medical images. Second, at the algorithm design level, researchers expect machines to learn to learn in a human way, that is, to classify and identify samples with a small number of samples, and to have the ability to quickly understand and generalize new concepts. In order to be able to learn with a limited amount of supervised information, research on small-sample learning (few-shot learning) has emerged. In small-sample classification, a model is trained on a set of classes with abundant samples, called base classes, and then trained and tested on another set of classes with only a few samples (new classes) where the classes do not intersect. Currently, there is a growing body of research work addressing few-shot learning, and with the development of deep learning, many novel few-shot learning methods have emerged. For example, self-supervised learning is used in the model characterization phase to better characterize the images; in the data expansion phase, small-sample learning tasks are handled by expanding from the original domain or expanding from the semantic space; in the learning phase, algorithms such as migration learning, metric learning, and meta-learning are used to better find a model with good generalization ability [24].

## 3. Method

### 3.1. Model Architecture

**Figure 3:**
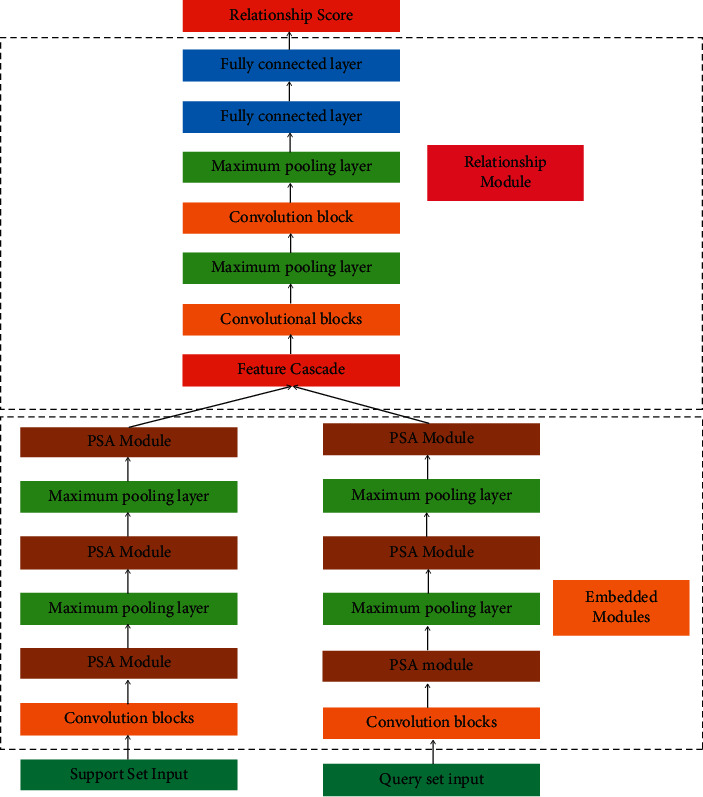
Model architecture diagram.

In this paper, based on convolutional neural networks and improved by small-sample learning, a new small-sample image classification algorithm is proposed by introducing a multiscale channel attention mechanism into the network in order to improve the accuracy of relational network image classification. Because the multiscale channel attention mechanism can focus on the important information in the sample feature space, the method can extract richer multiscale features of images and improve the classification results by relational metrics. The method can quickly and accurately detect the erroneous actions in physical education professional skills courses by batch normalizing the samples of erroneous actions in physical education teaching training and extracting the erroneous action features based on them, which can further improve the assessment standard of physical education professional skills courses. The proposed few-shot image classification network model based on multiscale channel attention mechanism is shown in Figure 3.

**Figure 4:**
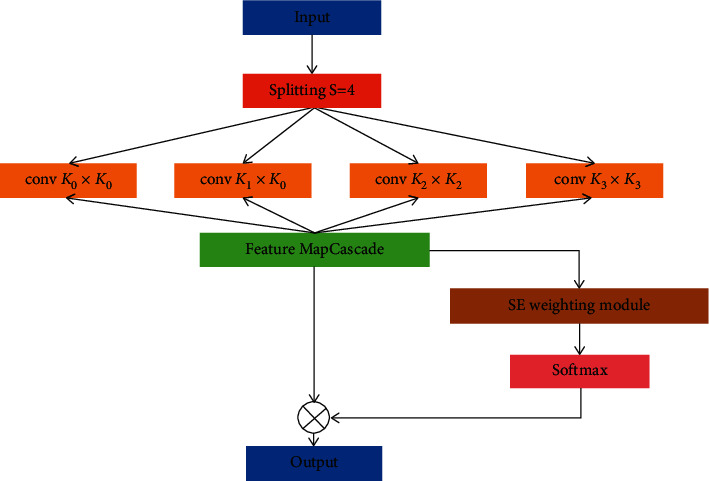
PSA module network structure.

The embedding module consists of one layer of convolutional blocks, three layers of PSA modules, and two layers of maximum pooling layers; the relationship module consists of two layers of convolutional blocks, two layers of maximum pooling layers, and two layers of fully connected layers. The obtained support set image features are cascaded with the query set image features and fed into the relational model. The PSA module network structure is a pyramidal cut attention module, and its network structure is shown in Figure 4. It divides the input feature maps into four scales and processes them in parallel with convolutional kernels of different sizes, where *K*_0_=3, *K*_1_=5, *K*_2_=7, *an*  *dK*_3_=9. In the figure, the SE weight module gets the channel attention first calculates the weights of different channel feature maps, then calibrates the weights by SoftMax layer, and finally gets the feature maps with different channel weights. In this paper, the PSA module is used to replace the last three layers of convolution blocks in the embedding module, which results in the improved embedding module (Figure 4).

### 3.2. Few-Shot Learning

**Figure 5:**
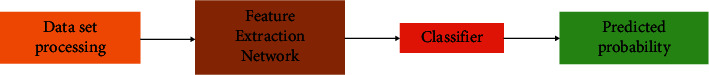
Few-shot image classification process.

Inspired by human intelligence, the purpose of studying small-sample learning is to hope that the model (algorithm) can act like a human brain, and after learning a large number of base classes, it only needs a small number of samples to quickly get new classes and acquire new knowledge to do the same thing. The few-shot image classification process (Figure 5) includes three parts: data set processing, feature extraction network, and classifier.

The basic model of few-shot learning is defined as *r*=*g*(*f*(·*|θ*)*|ω*), which consists of a feature extraction network *f*(·*|θ*) and a classifier *g*(·*|ω*), where 0 and *w* denote the parameters of *f* and *g*, respectively, *x* denotes the image to be recognized, *f*(·*|θ*) denotes the features extracted from image *x*, and *r* denotes the result of recognition of image *x*. In order to extract the effective features of the image, a feature extraction model is required for classification. For the model, the extracted image features should describe the image as effectively as possible so that the classifier can make better use of them. It can be seen that improving the image feature description and extraction will lead to better classification results. Attention mechanism, memory mechanism, etc. are common technical means for effective image feature extraction. Classifiers obtain different classes of objects by applying similarity measures to the features. Most of the classifiers used in few-shot image classification construct fully connected layers with SoftMax in the last layer of the convolutional neural network or apply *K*-nearest neighbor algorithm to the extracted image features, etc. The former is used for the classifier in this paper.

### 3.3. Convolutional Neural Networks

The pyramidal cut attention module (PSA) is a multiscale spatial and channel attention mechanism. The structure of the pyramid cut attention mechanism framework is shown in Figure 4. The input is converted into a multiscale feature map in the channel direction using a segmentation and concatenation module (SPC module). In particular, the implementation steps of the segmentation and concatenation module are as follows. The input feature map *X* is segmented into S parts, each represented by [*X*_0_, *X*_1_,…, *X*_*S*−1_] and the channel dimension *C′*, *C'* *=* *C*/*S*. A group convolution approach is introduced to process these parts separately in multiscale parallel. The SPC devises a new criterion to deal with the multiscale kernel and group size relationship.

(1) $$\begin{array}{c} G=2^{K-1/2}, \end{array}$$

where *G* is the group size and *K* is the kernel size. Moreover, when the kernel size *K* *=* *3, G* *=* *1*. The generating function of the multiscale feature map after the SPC module is as follows:

(2) $$\begin{array}{c} F_{i}=\text{conv}\left(k_{i}\times k_{i},G_{i}\right)\left(X_{i}\right),\;i=1,2,\text{…},S-1, \end{array}$$

where the size of the *i*-th kernel *k*_*i*_=2 × (*i*+1)+1 and the size of the *i*-th group *G*_*i*_=2^*K*_*i*_ − 1/2^. The obtained multiscale feature maps are stitched together as follows:

(3) $$\begin{array}{c} F=\text{cat}\left(\left[F_{0},F_{1},\text{…},F_{S-1}\right]\right). \end{array}$$

The attention weights of the feature maps at different scales are extracted using the SE weights module to obtain the attention vectors in the channel direction.

(4) $$\begin{array}{c} Z_{i}=\text{SEWeight}\left(F_{i}\right),\;i=1,2,\text{…},S-1,\\ Z=Z_{0}⊕Z_{1}⊕\ldots ⊕Z_{S-1}, \end{array}$$

where ⊕ is the cascade operator, *Z* is the attention weight of *F,* and *Z* is the multiscale attention weight vector. The SE module is the classical model in the channel attention mechanism, including Squeeze operation and Excitation operation. For any given transformation, the input *X* is mapped to the feature map *U*, where *U* ∈ *R*^*H*×*W*×*C*^. The feature *U* is first compressed to a 1 × 1 × *C* vector in spatial dimension by the Squeeze operation, that is, global average pooling. The Squeeze operation is given by the following equation:

(5) $$\begin{array}{c} z_{c}=F_{sq}\left(u_{c}\right)=\frac{1}{H\times W}\sum_{i=1}^{H}\sum_{j=1}^{W}u_{c}\left(i,j\right). \end{array}$$

The Squeeze operation is followed by the Excitation operation, which models the correlation between channels by forming a Bottleneck structure with two fully connected layers, generating a weight value for each channel and obtaining the set of individual channel weights. The attention vector in the channel direction is recalibrated using SoftMax to obtain the recalibrated attention weights for the multiscale channels.

(6) $$\begin{array}{c} {\text{att}}_{i}=\text{soft}\max\left(z_{i}\right)=\frac{\exp\left(z_{i}\right)}{\sum_{i=0}^{S-1}\exp\left(z_{i}\right)}, \end{array}$$

where SoftMax is used to obtain the recalibrated weights of the multiscale channels, which contain all the location information on the space and the attention weights in the channels. Finally, a refined feature map with richer multiscale feature information is obtained as the output.

(7) $$\begin{array}{c} \text{out }=\text{cat}\left(\left[Y_{0},Y_{1},\text{…},Y_{s-1}\right]\right). \end{array}$$

## 4. Experiment and Results

### 4.1. Experimental Setup

In order to demonstrate the effectiveness of the assessment and judgment model of physical education professional skills course based on convolutional neural network and few-shot learning, a class of students in a physical education college was used as the experimental object. Among them, the parameters of the deep convolutional neural network are set as shown in Table 1. The wrong actions in the process of physical education professional skills course assessment were detected by using the method of this paper (the detection method of deep convolutional neural network) and the action detection method of two-dimensional wavelet packet [16] and the action detection method of spatial clustering [17], respectively, and the effect of the three methods in detecting the wrong actions in physical education professional skills course assessment was compared under the condition that the number of experiments gradually increased. In the experimental process, the dataset used in this paper is a nonpublic dataset, and machine vision technology is used to capture physical education training movements, and the capture results are denoised and enhanced to improve the accuracy of the experimental results. On this basis, all experimental data are divided into two groups: one for deep convolutional neural network training and one for experimental testing.

All experiments in this section are done on a PC with NVIDIA GeForce RTX2080 8 GB graphics card, Intel i7-9700KF processor, and 16G running memory, using the Linux version of PyTorch deep learning framework to build the model. In this section, a four-layer convolutional Conv-64 will be used as the feature embedding network. An episode training mechanism is used to train the proposed network with a total of 3000 episodes, and each episode constructs a query set and a support set by sampling. For the 5-way (1-shot) few-shot classification task, 5 categories are drawn from each episode, and 1 support set sample and 15 query set samples are drawn from each class; then in each episode, a total of 80 samples are drawn in each episode. In the 5-way (5-shot) few-shot classification task, a total of 100 samples are extracted from each episode. The initial learning rate is 47, and the learning rate is halved for every 1000 episodes. 600 episodes are randomly sampled during the test to calculate the top-1 average accuracy, and the interval confidence level is set to 95%.

### 4.2. Comparison of Visualization Results

**Figure 6:**
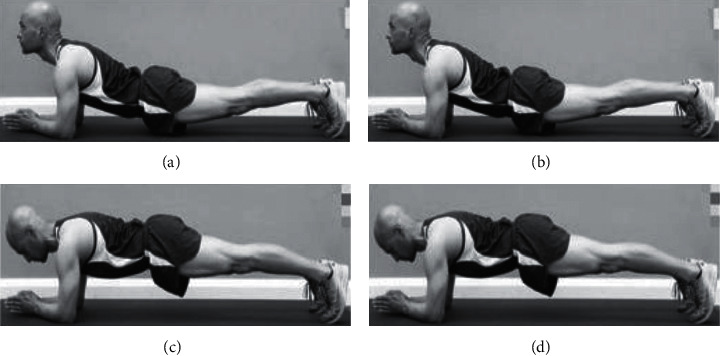
Results visualization. (a) Wrong action sample. (b) Recognition results of the proposed method. (c) Action recognition result of two-dimensional wavelet packets. (d) Action recognition result of spatial clustering.

Since several sports training error actions were involved in the experimental process, the detection results of different methods for one of the sports teaching training error actions are shown in Figure 6. Analysis of Figure 6 shows that Figure 6(a) is the standard sports teaching training process occurring error actions, including collapsing waist, lower back too low, lifting head, and collapsing hips. Different methods can detect the wrong actions of each part of sports personnel, and the detection results of this paper's method are highly consistent with Figure 6(a), while the method of action detection method of two-dimensional wavelet packet and the method of action detection method of spatial clustering have higher false detection rate when detecting wrong actions in physical education training, which indicates that the detection of erroneous movements in sports training is better with this paper's method. The detection effect of action detection method using two-dimensional wavelet packet and action detection method using spatial clustering is better than the detection effect of action detection method using two-dimensional wavelet packet.

### 4.3. Performance Comparison

On the basis of relevant experiments, three methods were used to detect sports errors in the experimental samples, and the error rates of sports error detection of different methods were compared, and the results of the comparison were used to measure the comprehensive effectiveness of the three different methods in detecting errors in physical education training, and the comparison results are shown in Table 2.

Analysis of Table 2 shows that, with the increasing number of experiments, the error rate of the detection error rate of the paper's method for physical education training errors has been maintained at a low level. The average error rate of the detection of wrong movements of physical education training of this paper is about 0.034%, while the average error rate of the action detection method of two-dimensional wavelet packet is about 0.103% and the average error rate of the action detection method of spatial clustering is about 0.168%, which shows that the detection accuracy of this paper is higher. When using this paper method to detect the wrong movements of physical education training, the error can be controlled within a reasonable area. In order to verify the effectiveness and robustness of this paper's method in the detection of erroneous movements in physical education training, four test indexes, ACC (accuracy), TPR (sensitivity), FPR (specificity), and PPV (positive prediction rate), were taken for quantitative comparison, and the specific formulae are described as follows:

(8) $$\begin{array}{c} \text{ACC}=\frac{\text{TP}+\text{TN}}{\text{TP}+\text{TN}+\text{FP}+\text{FN}},\\ \text{TPR}=\frac{\text{TP}}{\text{TP}+\text{FN}}\text{,}\\ \text{FPR}=\frac{\text{TP}}{\text{TN}+\text{FP}}\text{,}\\ \text{FPR}=\frac{\text{TP}}{\text{TN}+\text{FP}}\text{,} \end{array}$$

where TP indicates the number of samples judged to be positive and in fact positive; FP indicates the number of samples judged to be positive but in fact negative; TN indicates the number of samples judged to be negative and in fact negative; and FN indicates the number of samples judged to be negative but in fact positive. The higher the ACC and TPR, and the lower the FPR and PPV, the better the detection performance.  Table 3 shows the test experimental results of the detection of erroneous movements in physical education training. From Table 3, it can be obtained that among the three methods of physical education training error action detection, the four parameter indexes of ACC, FPR, PPV, and TPR of this paper's method are better than the other two methods, in which the detection accuracy of this paper's method reaches more than 98%, which verifies the superior performance of deep convolutional neural network.

## 5. Conclusion

In the process of assessing and judging the physical education professional skills course, after students first learn the movements, the movement process is often accompanied by wrong movements because the connection between the nerves and muscles of the movement subject is not yet precise, so how to correct and prevent wrong movements in the process of assessing and judging the physical education professional skills course is a basic requirement for physical education teachers to improve the quality of teaching sports technology. According to relevant research, it is known that in the past, the process of correcting the movements in the assessment and judging of physical education professional skills courses could not accurately detect the wrong movements, so it was not possible to correct the wrong movements in time. Under the impetus of the international sports mega-trend, various sports activities have continuously entered people's vision, and people began to pay attention to the training of various sports activities. In the actual physical education professional skills course assessment and judging process, the training of sports personnel need to be based on the standard movements of various sports activities, but different people have different levels of understanding of various sports, and some sports personnel have less developed motor nerves and often make action errors when conducting physical education professional skills course assessment and judging, and it is difficult to achieve a quick grasp of the correct action in this situation. In this case, it is required that the teaching staff can effectively point out and correct the wrong movements of the athletes in the process of assessing and judging the physical education professional skills courses. Therefore, this paper proposes a convolutional neural network and small-sample learning method for detecting incorrect movements in physical education professional skills assessment, and proves that this method can effectively detect the incorrect movements of athletes in the process of physical education professional skills assessment. It can effectively control the detection error and judge the wrong action timely and accurately. In the future application of the method in this paper, we need to focus on the analysis of training sample data and use other methods to improve the detection speed, so as to give strong technical support to the detection of erroneous movements in the assessment and judgment of physical education professional skills courses.

In the future, in order to further improve the performance, we plan to conduct research on meta-learning and graph convolution for the assessment and judging model of physical education professional skills courses.

## Figures and Tables

**Table 1:** Parameter setting of deep convolutional neural network.

Number of layers	Function
1	Input layers
2	Convolutional layers 3–64
3	Convolutional layers 3–64
4	Maximum pooling layer
5	Convolutional layer 3–128
6	Convolutional layer 3–128
7	Maximum pooling layer
8	Convolutional layer 3–256
9	Convolutional layer 3–256
10	Maximum pooling layer
11	Convolutional layer 3–512
12	Convolutional layer 3–512
13	Maximum pooling layer
14	Convolutional layer 3–512
15	Convolutional layer 3–512
16	Fully connected layer 3072
17	Fully connected layer 1024
18	Output layer

**Table 2:** Comparison of the detection error rate of different methods.

Number of experiments (times)	Error rate (%)		
	Methodology of this paper	Two-dimensional wavelet packet	Spatial clustering
15	0.021	0.078	0.15
25	0.019	0.089	0.143
35	0.045	0.153	0.249
45	0.013	0.124	0.199
55	0.032	0.086	0.132
65	0.075	0.088	0.133

**Table 3:** Detection results of wrong movements in the assessment of physical education professional skills courses.

Test index	Methodology of this paper	Two-dimensional wavelet packet	Spatial clustering
ACC	0.984	0.853	0.836
TPR	0.954	0.854	0.853
PPV	0.092	0.254	0.236
FPR	0.017	0.042	0.117

## Data Availability

The datasets used in this paper are available from the corresponding author upon request.
